# Editorial on “Application of LiDAR Remote Sensing and Mapping”

**DOI:** 10.3390/s26154910

**Published:** 2026-08-04

**Authors:** Xiaoxiao Zhu, Sheng Nie, Cheng Wang

**Affiliations:** 1 State Key Laboratory of Earthquake Dynamics and Forecasting, Institute of Geology, China Earthquake Administration, Beijing 100029, China; zhuxx@ies.ac.cn; 2 Key Laboratory of Seismic and Volcanic Hazards, Institute of Geology, China Earthquake Administration, Beijing 100029, China; 3 Aerospace Information Research Institute, Chinese Academy of Sciences, Beijing 100094, China; 4 School of Land Science and Technology, China University of Geosciences, Beijing 100083, China

## 1. Introduction

Light Detection and Ranging (LiDAR) has become one of the most important technologies in modern remote sensing and mapping [1,2]. By actively emitting laser pulses and measuring the returned signals, LiDAR systems provide accurate, high-resolution, and three-dimensional observations of natural and built environments, including terrain, vegetation, buildings, and infrastructure [3,4]. Compared with conventional passive optical imagery, LiDAR point clouds directly capture geometric structure and elevation information, making them particularly valuable for applications requiring precise spatial reconstruction, structural characterization, and object-level interpretation.

In recent years, LiDAR remote sensing has expanded rapidly across multiple platforms, including terrestrial laser scanning, mobile laser scanning, airborne laser scanning, UAV-borne LiDAR, and spaceborne laser altimetry [1,5]. These platforms have substantially broadened the spatial scales and application scenarios of LiDAR-based mapping, ranging from indoor building modelling and urban infrastructure inspection to forest parameter retrieval, geological outcrop mapping, highway reconstruction, and ecological monitoring [6,7]. The increasing availability of dense point clouds has introduced new methodological challenges, including point-cloud registration, semantic segmentation, feature extraction, data compression, multi-source data fusion, and uncertainty assessment. Meanwhile, the growing volume and complexity of LiDAR data have placed increasing demands on computational efficiency.

Advances in artificial intelligence, particularly machine learning and deep learning, together with developments in high-performance computing, have further accelerated the transformation of LiDAR data from geometric observations into application-oriented knowledge [8,9]. Central research topics include point cloud classification, object detection, surface reconstruction, and 3D scene understanding, while simultaneous localization and mapping (SLAM) remains a key research direction for mobile and autonomous mapping systems [10]. In addition, the integration of LiDAR with optical imagery, global navigation satellite system data, inertial measurement units, hyperspectral data, and other satellite observations has created new opportunities for accurate and scalable mapping. These advances are reshaping practical workflows in forestry, transportation, urban planning, geology, cultural heritage conservation, environmental monitoring, and intelligent infrastructure management.

This Special Issue, entitled “Application of LiDAR Remote Sensing and Mapping”, was organized to bring together recent advances in LiDAR sensing, point cloud processing, and mapping applications. The papers published in this Special Issue cover a broad spectrum of topics that can be grouped into four thematic areas: (i) data acquisition and system development, including mobile mapping, terrestrial laser scanning, and LiDAR system and sensor development; (ii) forestry and ecological applications, such as forest inventory, tree-trunk detection, individual-tree DBH estimation, and biomass estimation; (iii) the built environment and infrastructure, encompassing indoor segmentation, tunnel leakage detection and semantic segmentation, highway reconstruction, and traditional building material classification; and (iv) data processing and analysis, including geological classification, depth image reconstruction, point cloud compression, feature selection, and other point cloud applications. Collectively, these studies demonstrate the versatility of LiDAR technologies and highlight the growing importance of robust algorithms, multi-source data integration, and application-driven validation in the development of next-generation remote sensing and mapping systems.

This Editorial summarizes the major contributions of the papers published in this Special Issue and discusses emerging trends and future research directions in LiDAR remote sensing and mapping.

## 2. Overview of Published Papers

The study by Chen et al. (contribution 1) addresses the problem of highway reconstruction using mobile laser scanning data. Highways are complex transportation environments containing roads, markings, guardrails, traffic signs, vegetation, and other infrastructure elements. Accurate semantic understanding of such environments is essential for intelligent transportation systems and infrastructure management. The authors propose a fine-grained semantic segmentation and reconstruction method based on dense 3D point clouds. By combining multi-scale object-based data augmentation, down-sampling strategies, and a KPConv-based deep learning network, the proposed framework achieves effective segmentation of 27 highway-related categories. The reconstructed highway environment demonstrates the practical value of LiDAR point clouds for digital infrastructure modelling and transportation management.

Zhao et al. (contribution 2) focus on high-accuracy online mapping in dynamic environments. Dynamic objects such as vehicles, cyclists, and pedestrians often degrade the quality of LiDAR-based mapping, especially in autonomous driving and robotic navigation scenarios. The authors propose a framework that integrates weight matching LiDAR-IMU-GNSS odometry with object-level dynamic point cloud filtering based on a pseudo-occupancy grid. The odometry component incorporates IMU pre-integration, ground segmentation, motion compensation, and feature matching based on geometric and reflectance similarities. The dynamic filtering strategy improves map clarity by identifying and removing highly dynamic point cloud clusters. This work contributes to robust online mapping under complex and changing environmental conditions.

Liu et al. (contribution 3) present MC-H-Geo, a multi-scale contextual hierarchical framework for fine-grained lithology classification from terrestrial laser scanning point clouds. Lithological mapping of geological outcrops is important for reservoir characterization and geological interpretation, but subtle differences among rock types can be difficult to identify using geometric information alone. The proposed framework integrates multi-scale contextual features, hierarchical expert classification, and geological post-processing to improve classification accuracy and spatial consistency. The study demonstrates the potential of LiDAR point clouds for geological mapping while also emphasizing the need for multi-sensor data fusion to overcome the limitations of TLS-only data in complex geological scenes.

Zou et al. (contribution 4) investigate the influence of sampling density on indoor building LiDAR point-cloud segmentation. Although dense point clouds can improve geometric representation, excessive density increases memory use and computational cost. The authors establish a unified evaluation framework in which sampling density is treated as the key independent variable. Three representative deep learning backbones, enhanced with a Point Transformer module, are evaluated under a standardized protocol. The results reveal an engineering-feasible density range that balances segmentation accuracy and efficiency, providing useful guidance for scan planning, indoor mapping, and Scan-to-BIM workflows. This contribution is particularly valuable because it links algorithmic performance with practical data acquisition strategies.

Zhang et al. (contribution 5) propose a leakage detection method for subway tunnels using mobile laser scanning point clouds. Leakage detection is a challenging task because tunnel point clouds contain noise, complex geometric structures, and intensity variations. The authors integrate intensity features and geometric features with an XGBoost classifier. The workflow includes noise filtering using RANSAC, intensity-based candidate extraction, geometric feature calculation, neighborhood-scale optimization, and binary classification. The method achieves strong performance across heterogeneous datasets, demonstrating the value of combining radiometric and geometric information for infrastructure defect detection. This study illustrates the growing role of LiDAR in underground infrastructure inspection and maintenance.

Lovrinčević et al. (contribution 6) examine new possibilities for field data survey in forest road design. Forest road planning requires accurate spatial data, but traditional field surveying methods may be labor-intensive and less efficient in complex terrain. The authors compare classical surveying, modern surveying, UAV photogrammetry, and LiDAR-based data. Their results show that modern remote sensing approaches can provide high-accuracy spatial information for forest road design, although vegetation, biomass, and terrain conditions remain important sources of uncertainty. This work demonstrates how LiDAR and related surveying technologies can improve forest engineering, road planning, and operational decision-making.

Yueliang et al. (contribution 7) investigate aboveground biomass estimation in Grain for Green Program stands by integrating UAV-LiDAR and Sentinel-2 data. Aboveground biomass is a key indicator for evaluating forest health, carbon sink capacity, and ecological restoration outcomes. The authors construct a high-quality biomass sample dataset using UAV-LiDAR data and tree species information, and then estimate biomass using Sentinel-2 (Friedrichshafen, Germany) imagery and a gradient-boosting decision tree algorithm. The study highlights the complementarity of LiDAR-derived structural information and satellite-based spectral information. This contribution provides an effective framework for regional biomass estimation and ecological assessment in restoration-oriented forest stands.

Goldfisher et al. (contribution 8) report a gain-switched Ho:YAP laser operating at 2117 nm for laser distance measurement and remote sensing applications. The laser produces short pulses with nanosecond-scale duration and operates within a short-wavelength infrared atmospheric transmission window. Such laser sources are relevant to LiDAR systems because pulse duration, pulse energy, wavelength, compactness, and energy efficiency directly affect ranging performance, spatial resolution, and environmental sensing capability. This study contributes to the hardware and sensor-development dimension of LiDAR remote sensing by advancing laser source design for precise distance measurement and environmental gas detection.

Ma et al. (contribution 9) propose an improved cylinder-based tree trunk detection method for forestry applications. Accurate trunk detection is essential for extracting individual tree parameters and supporting forest inventory. Two-dimensional trunk detection methods can lose spatial information, while conventional cylinder fitting can be sensitive to parameter selection in complex forest environments. The authors develop an improved Random Sample Consensus Cylinder Fitting approach optimized for detecting cylindrical tree trunks in LiDAR point clouds. The method improves accuracy, robustness, and adaptability in forest scenes, providing an effective tool for individual tree extraction and forest structural analysis.

Wu et al. (contribution 10) explore the classification of traditional building materials in Southern Fujian using reflection intensity values from ground-based LiDAR. Traditional buildings often contain diverse materials such as wood, stone, and brick, and their digital documentation is important for cultural heritage conservation. The authors use LiDAR intensity information, distance calibration, noise removal, and polynomial regression to support automated material classification. Indoor experiments and outdoor case studies validate the approach. This study expands the use of LiDAR beyond geometric reconstruction by demonstrating how intensity information can support semantic interpretation and heritage-oriented material analysis.

Yang et al. (contribution 11) develop a depth image reconstruction algorithm using a two-dimensional Kaniadakis entropy threshold for photon-counting LiDAR. Photon-counting LiDAR can achieve high sensitivity, but its imaging quality is often affected by background noise, especially under low signal-to-background ratio conditions. The proposed method transforms weak-signal extraction into a denoising problem by exploiting signal aggregation and spatiotemporal correlation in point cloud-intensity data. Simulations and outdoor experiments demonstrate improved target recovery under challenging noise conditions. This work advances LiDAR imaging and reconstruction methods for low-signal and high-background environments.

Dumic and da Silva Cruz (contribution 12) provide a meta-survey of three-dimensional point cloud applications, datasets, and compression methodologies for remote sensing. As point cloud data become increasingly large and widely used, efficient storage, transmission, and processing are becoming critical issues. The review covers point cloud applications in remote sensing, available datasets, and compression methods ranging from classical tree- and projection-based techniques to recent deep learning approaches. This contribution offers a comprehensive overview of the state of the field and identifies emerging challenges and opportunities for future point cloud research.

Li and Jia (contribution 13) develop an individual-tree diameter at breast height (DBH) estimation framework using airborne LiDAR data. Field-based DBH measurements are accurate but labor-intensive, while airborne LiDAR-derived variables are often high-dimensional and redundant. The authors combine a multi-stage feature selection (MSFS) strategy integrating Pearson correlation analysis, mutual information, and Boruta with XGBoost. From 104 LiDAR-derived and structural variables, the framework identifies an optimized feature subset and achieves an R^2^ of 0.901 and an RMSE of 1.647 cm, outperforming decision tree, random forest, and gradient-boosting alternatives. This contribution demonstrates how systematic feature optimization can improve the accuracy and stability of forest structural parameter retrieval and supports refined individual-tree inventory and large-scale forest monitoring.

Tong et al. (contribution 14) propose MFPNet for fine-grained semantic segmentation of regular tunnel point clouds. Tunnel scenes contain structures with large scale differences, indistinct boundaries, and strongly imbalanced categories, making small components difficult to recognize. The network integrates multi-scale kernel point convolution, an error-feedback-based local-global feature fusion module, and adaptive channel-spatial feature enhancement to strengthen cross-scale contextual representation. Experiments report an mIoU of 87.5% and an overall classification accuracy of 96.3%, with substantial improvements over representative point-based networks. This work advances automated tunnel scene understanding and provides technical support for tunnel digital twins, structural health assessment, and intelligent inspection.

Collectively, these 14 contributions reflect the breadth and depth of current research in LiDAR remote sensing and mapping. They show that LiDAR is not a single-purpose technology but a flexible sensing framework that supports geometric reconstruction, semantic interpretation, environmental monitoring, infrastructure inspection, forest inventory and individual-tree parameter estimation, geological analysis, cultural heritage documentation, and advanced sensor development. The collection also demonstrates that future progress in this field will depend on the close integration of sensor hardware, point cloud algorithms, multi-source data fusion, domain knowledge, and real-world validation.

## 3. Conclusions

The papers collected in this Special Issue demonstrate the rapid evolution of LiDAR remote sensing and mapping, spanning data acquisition, intelligent interpretation, and application-driven decision making. The studies cover both methodological advances and practical applications, including semantic segmentation, online mapping, lithology classification, indoor building segmentation, tunnel leakage detection, tunnel point-cloud semantic segmentation, forest road survey, biomass estimation, individual-tree DBH estimation, laser source development, tree trunk detection, traditional building material classification, depth image reconstruction, and point cloud compression.

Several key trends can be identified from this collection. First, LiDAR point clouds are increasingly being combined with machine learning and deep learning methods to achieve automated interpretation of complex three-dimensional scenes. Second, application-specific validation is becoming essential: algorithms are no longer evaluated only by generic accuracy metrics, but also by their performance in highways, tunnels, forests, buildings, geological outcrops, and heritage sites. Third, multi-source data fusion is emerging as a critical direction, especially for tasks in which geometry alone is insufficient. The integration of LiDAR with optical imagery, satellite data, inertial sensors, GNSS, and domain-specific prior knowledge can improve robustness, scalability, and interpretability. Fourth, computational efficiency, sampling density, and data compression are becoming central concerns as LiDAR systems generate increasingly dense and large-scale point clouds.

Looking forward, several research directions warrant further investigation. Robust semantic segmentation across heterogeneous scenes remains a major challenge, particularly when models trained in one region, platform, or environment are applied elsewhere. Domain adaptation, self-supervised learning, foundation models for point clouds, and uncertainty-aware inference may offer promising solutions. In forest and ecological applications, the integration of UAV-LiDAR, airborne LiDAR, spaceborne LiDAR, and multispectral satellite data will be important for multi-scale monitoring of vegetation structure, biomass, carbon storage, and ecological restoration. In urban and infrastructure applications, LiDAR-based digital twins, automated defect detection, and real-time mapping will play an increasingly important role in transportation, underground infrastructure, and smart city management. In addition, advances in LiDAR hardware, photon-counting imaging, laser source design, and data compression will continue to influence the accuracy, efficiency, and deployability of future systems.

We would like to express our sincere gratitude to all authors who contributed their valuable research to this Special Issue. Their studies have advanced the field of LiDAR remote sensing and mapping and have demonstrated the diverse scientific and practical potential of LiDAR technologies. We also thank the reviewers for their careful evaluations, constructive comments, and professional support, which have helped improve the quality and clarity of the published papers. Finally, we acknowledge the editorial team of *Sensors* for their continuous assistance throughout the preparation and publication of this Special Issue.

We hope this collection provides readers with useful insights into recent advances in LiDAR remote sensing and mapping, and stimulates further research on intelligent point cloud processing, multi-platform LiDAR observation, sensor development, and application-oriented 3D mapping.

## References

[1] Wehr A., Lohr U. (1999). Airborne laser scanning—An introduction and overview. ISPRS J. Photogramm. Remote Sens..

[2] Baltsavias E.P. (1999). Airborne laser scanning: Basic relations and formulas. ISPRS J. Photogramm. Remote Sens..

[3] Vosselman G., Maas H.-G. (2010). Airborne and Terrestrial Laser Scanning.

[4] Shan J., Toth C.K. (2018). Topographic Laser Ranging and Scanning: Principles and Processing.

[5] Mallet C., Bretar F. (2009). Full-waveform topographic lidar: State-of-the-art. ISPRS J. Photogramm. Remote Sens..

[6] Wulder M.A., Coops N.C., Hudak A.T., Morsdorf F., Nelson R., Newnham G., Vastaranta M. (2013). Status and prospects for LiDAR remote sensing of forested ecosystems. Can. J. Remote Sens..

[7] Dubayah R., Blair J.B., Goetz S., Fatoyinbo L., Hansen M., Healey S., Hofton M., Hurtt G., Kellner J., Luthcke S. (2020). The Global Ecosystem Dynamics Investigation: High-resolution laser ranging of the Earth’s forests and topography. Sci. Remote Sens..

[8] Qi C.R., Su H., Mo K., Guibas L.J. PointNet: Deep learning on point sets for 3D classification and segmentation. Proceedings of the IEEE Conference on Computer Vision and Pattern Recognition.

[9] Qi C.R., Yi L., Su H., Guibas L.J. PointNet++: Deep hierarchical feature learning on point sets in a metric space. Proceedings of the 31st International Conference on Neural Information Processing Systems.

[10] Zhang J., Singh S. LOAM: Lidar odometry and mapping in real-time. Proceedings of the Robotics: Science and Systems.

